# A systematic review of therapeutic options for lymphocytic esophagitis

**DOI:** 10.1093/dote/doaf112

**Published:** 2025-12-05

**Authors:** Bachviet Nguyen, Chun Fang Cheng, Fahd Jowhari

**Affiliations:** Department of Medicine, University of British Columbia, 13750, 96 Ave. Critical Care Tower, 3rd Floor, Surrey, British Columbia, V3V 1Z2, Canada; Department of Statistics, University of British Columbia, 3182 Earth Sciences Building, 2207 Main Mall, Vancouver V6T 1Z4, Canada; Division of Gastroenterology, University of British Columbia Okanagan, 564 Leon Avenue, Kelowna, V1Y 6J6, Canada

**Keywords:** diseases of the esophagus, proton pump inhibitors (PPIs), lymphocytic esophagitis, budesonide, biologics, tacrolimus, esophagitis

## Abstract

**Background:**

Lymphocytic esophagitis (LyE) is a novel rare esophageal disorder characterized by intraepithelial lymphocytic infiltration of the esophagus in a peripapillary distribution, without the involvement of granulocytes. The optimal treatment strategy for this condition remains uncertain. We aimed to synthesize the current evidence for the treatment of lymphocytic esophagitis.

**Methods:**

We performed a systematic review according to PRISMA guidelines, searching MEDLINE, Embase, and Google Scholar. Studies with non-primary data or insufficient treatment data were excluded. Descriptive statistics were performed on patient demographics and treatment outcomes.

**Results:**

Thirty nine articles from 2012–2024 were included (154 patients total). Proton pump inhibitors (PPIs) were the most common initial therapy for LyE (*n* = 65), followed by topical steroids (*n* = 23). A greater proportion of patients experienced a symptomatic, endoscopic, and histologic response from the initial use of topical steroids as monotherapy or part of combination therapy (with PPIs) compared to PPIs alone. Symptomatic recurrence was more common after initial use of topical steroids compared to PPIs. Balloon dilation was effective in relieving symptomatic esophageal dysphagia. Other therapies included biologics, endoscopic botulinum injections, sucralfate, and tacrolimus. The average follow-up duration was 8.98 months.

**Conclusions:**

For patients with LyE, topical steroids seem to provide greater symptomatic and histologic benefit compared to PPIs, although recurrence is more common. For patients not already on acid suppression therapy, PPIs may still be a reasonable first-line option, especially when prioritizing safety. Further prospective studies are needed to formally assess the comparative safety and efficacy of the various treatment modalities, including novel immunosuppressive therapies.

## INTRODUCTION

Lymphocytic esophagitis (LyE) is a novel rare esophageal disorder involving intraepithelial lymphocytic infiltration of the esophagus in a peripapillary distribution without the involvement of granulocytes, leading to epithelial damage.^1^ Although many years have passed since it was first described by Rubio *et al*.^2^ in 2006, the pathophysiology, diagnostic criteria, and treatment of this disorder remains controversial, including whether it is a truly distinct entity or a manifestation of other disorders. Previous studies have hypothesized that LyE may be a result of topical injury, hypersensitivity reaction, or a primary autoimmune process.^3^^,^^4^

Patients with LyE most commonly experience esophageal dysphagia, gastroesophageal reflux, bolus impaction, chest pain, and abdominal pain. Associated complications include esophageal perforation and esophageal motility disorders.^5^ Endoscopy may reveal esophageal strictures, rings, furrows, and exudates.^1^

Currently, there are no standard treatment guidelines for LyE and patients are often treated empirically in a manner similar to that of eosinophilic esophagitis (EoE).^5^ To date, no systematic reviews or randomized control trials have been published on the treatment of lymphocytic esophagitis. This study aims to systematically synthesize the current state of the evidence on the treatment of this disorder to help clinicians manage this poorly understood condition.

## METHODS

A systematic review was performed according to PRISMA 2020 guidelines.^6^ This study was not prospectively registered. MEDLINE, Embase, and Google Scholar were searched from inception to May 30, 2024 using terms related to the treatment of lymphocytic esophagitis (Appendix SA). The titles and abstracts of articles were screened by two independent reviewers (BN, FJ) to remove duplicates and studies that did not meet inclusion criteria. We included case reports, case series and cohort studies. For an article to be included, it had to (i) Report patients with histologically confirmed LyE, (ii) Explicitly include a histologic definition of LyE, and (iii) Include primary data on the treatment of lymphocytic esophagitis as well as responses to treatment.

We excluded studies with non-primary data (i.e. reviews, commentaries), non-human data, studies whose full-texts that could not be acquired (with the exception of conference abstracts), and studies lacking data on treatment outcomes of LyE.

**Fig. 1:**
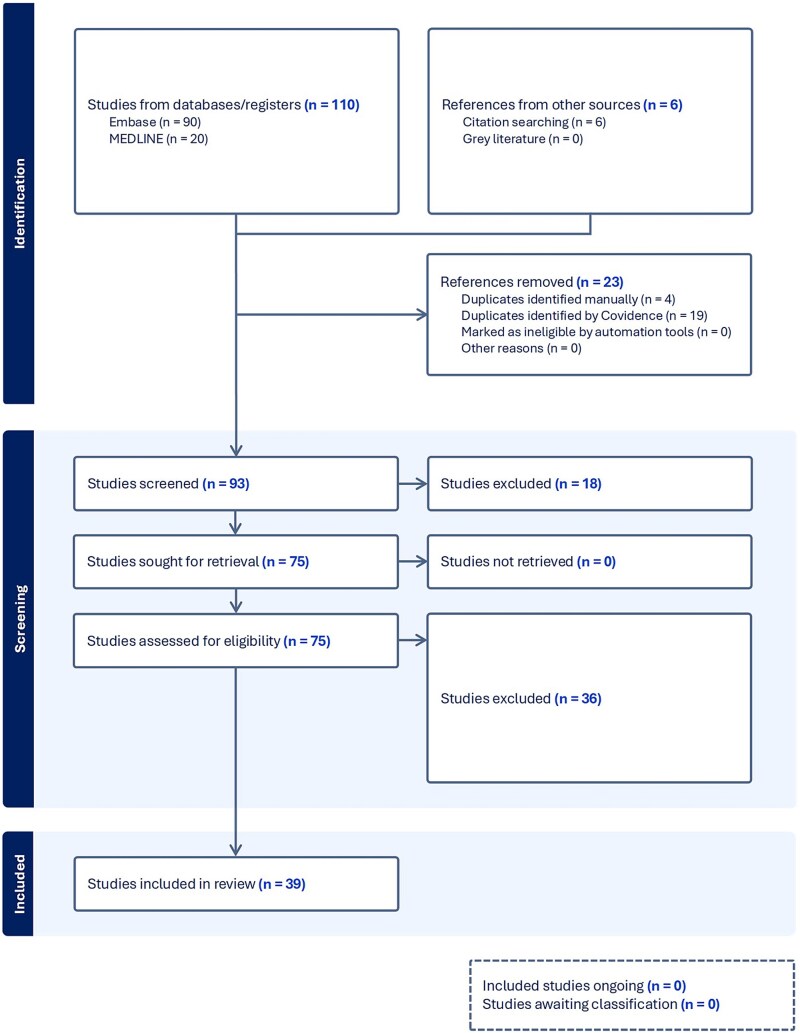
PRISMA flow diagram, where ‘n’ represents the number of studies.

Following initial screening, two independent reviewers (BN, CFC) performed abstract screening, a full text review to further remove ineligible articles, and extracted data on variables of interest (Fig.  1). Discussion between with a third reviewer (FJ) was used to decide whether an article was ultimately included for analysis. Quality analysis of each study was performed according to the Joanna–Briggs Institute Critical Appraisal Checklist for Case Reports/Case Series (Appendix SC).

For the purposes of our study, symptomatic response was defined as the patient self-reporting any improvement in the symptoms, while endoscopic response was defined as any improvement in the gross appearance of the esophagus on repeat endoscopy. Histologic response was defined as any improvement in the microscopic appearance of repeat biopsy samples.

### Statistical analysis

Data on patient demographics, past medical history, symptoms, endoscopy findings, biopsy results, treatments, and response to treatment was collected and pooled for analysis. Given that the studies included in this systematic review consisted of retrospective case reports and small case series, only descriptive statistics could be performed (frequencies, numbers, and means) instead of a formal meta-analysis. Heterogenous reporting of outcomes (i.e. no odds ratios for each outcome that could be pooled) across studies, as well as inability to manually calculate odds ratios based on the data available also precluded a meta-analysis.

## RESULTS

A total of 39 articles (35 case reports, 4 case series) from 2012–2024 were selected for analysis (Appendix SA).

### Demographics

A total of 154 patients were included in our study. Most of the studies took place in the United States (27/39), followed by the European continent (7/39) and Australia (3/39). One case report each came from Canada and Japan, respectively.^7^^,^^8^ The mean patient age at diagnosis was 56.2 years old (range 24–89) with 61 male patients (39.6%) and 93 female patients (60.4%). Most of the studies did not explicitly mention patient ethnicity; 23 patients were identified as Caucasian and 15 were non-Caucasian, 4 of which were of African descent (Appendix SB, Table  B.1) .

### Comorbidities and past medical history

23 patients in our study had inflammatory bowel disease prior to diagnosis with LyE (14.8%). Other common comorbidities included rheumatoid arthritis (*n* = 12; 7.8%), thyroid disease (*n* = 10; 6.5%), gastroesophageal reflux disease (GERD) (*n* = 22; 14.2%), and tobacco use (*n* = 21; 13.6%). Thirty six patients (23.4%) were on prior acid suppression with only a proton inhibitor (PPI) and 1 patient was on both a PPI and H2 receptor antagonist prior to diagnosis with LyE. In two studies, the development of LyE was associated with the use of Nivolumab for treatment of renal cell carcinoma^9^^,^^10^ (Appendix SB, Table  B.1).

### Symptoms

Dysphagia was the most common presenting symptom (*n* = 106; 68.8%), followed by heartburn (*n* = 39; 25.3%), abdominal pain (*n* = 19; 12.3%), nausea/vomiting (*n* = 14; 9.1%), odynophagia (*n* = 12; 7.8%), weight loss (*n* = 10; 6.5%), chest pain (*n* = 8; 5.2%), and bolus impaction (*n* = 6; 3.9%). Five patients initially presented with esophageal perforation (3.2%). Eleven patients were asymptomatic and diagnosed incidentally (7.1%) (Appendix SB, Table  B.2).

### Endoscopic findings

Abnormal esophageal findings on upper endoscopy mainly consisted of inflammation (*n* = 39; 25.3%), rings (*n* = 36; 23.4%), stricture (*n* = 36; 22.7%), exudate (*n* = 16; 10.4%), and plaques (*n* = 4; 2.6%). Forty six patients (29.9%) had a normal appearing mucosa on endoscopy while 4 also had findings of GERD (2.6%) (Appendix SB, Table  B.2).

### Initial treatment

Data for any response to treatment was available for 82 patients (Appendix SB, Table  B.6). PPIs were overall the most common initial therapy for LyE (*n* = 65), followed by topical steroids (*n* = 23). A greater proportion of patients experienced a symptomatic benefit, endoscopic response, and histologic response from the initial use of topical steroids as a monotherapy or part of combination therapy (with PPIs) compared to PPIs alone (Table 1). Symptomatic recurrence was more common after initial use of topical steroids as a monotherapy or part of combination therapy compared to PPIs alone in patients who initially responded to treatment (Table 1). 84.6% (11/13) of patients who initially underwent endoscopic dilation experienced symptomatic improvement and 57.1% (4/7) had their esophageal strictures successfully treated, but 28.6% (2/7) of these patients experienced symptomatic recurrence. In Schoepfer *et al*.^11^ tacrolimus was used as an initial therapy which was associated with symptomatic improvement in 57.1% (4/7) of patients and an endoscopic response in 42.9% (3/7) of patients. Biologics were used in Lee *et al*.^12^ which led to a symptomatic improvement in 5/6 patients, endoscopic improvement in 57.1% (4/7) of patients, and histologic improvement in 50% (3/6) of patients. 2/3 (100%) of patients in our review did not receive any treatment but experienced spontaneous resolution of their symptoms, however the patient in Lee *et al*.^12^ did not have histologic improvement after having no intervention. Side effects of PPIs, steroids, biologics, or tacrolimus were not discussed in the papers we reviewed (Appendix SB, Table  B.3).

**Table 1:** Initial response to treatment and symptomatic recurrence

**Initial treatment**	**Symptomatic response n/N (%)**	**Endoscopic response n/N (%)**	**Histologic response n/N (%**)	**Symptomatic recurrence n/N (%)**
**PPI monotherapy**	19/45 (42.2%)	4/17 (23.5%)	4/13 (30.8%)	1/5 (20%)
**Steroid monotherapy**	11/11 (100%)	7/12 (58.3%)	6/6 (100%)	2/4 (50%)
**PPI + Steroid**	6/9 (66.7%)	2/6 (33.3%)	2/7 (28.6%)	2/3 (66.7%)
**Dilation part of combination therapy**	11/13 (84.6%)	4/7 (57.1%)	0/2 (0%)	2/7 (28.6%)
**PPI monotherapy or combination therapy**	31/62 (50.0%)	7/20 (35.0%)	6/21 (28.6%)	4/13 (30.8%)
**Steroid monotherapy or combination therapy**	19/23 (82.6%)	10/19 (52.6%)	8/13 (61.5%)	4/8 (50%)

### Second-line treatment

Topical steroids were the most common second-line treatment for patients who initially were treated with acid suppression (*n* = 7), with 33.3% (1/3) of patients having a symptomatic response. For patients with esophageal strictures, endoscopic balloon dilation was used (*n* = 5), with 50% (3/6) of patients experiencing symptomatic benefit. One patient received esophageal Botox injections with improvement of symptoms, while another received famotidine and itopride in addition to their PPI with no response^13^^,^^14^ (Appendix SB, Table  B.4).

### Third-line treatment

One patient received Vedolizumab which led to a symptomatic and histologic response, after previously receiving a PPI and oral Budesonide as an unsuccessful first and second-line treatment, respectively.^15^ In Pizzuti *et al*.^10^ sucralfate in addition to a PPI was used which also improved symptoms (Appendix SB, Table  B.5).

The average follow-up duration was 8.98 months, with 18 patients receiving repeat endoscopy as part of the follow-up evaluation.

## DISCUSSION

This study represents the first systematic review on the treatment of lymphocytic esophagitis incorporating evidence from 154 patients across 39 publications. Currently, there are no standard treatment guidelines for this rare and poorly understood disorder.

The reported demographic profile was predominantly composed of adult, middle-aged women from developed countries, a trend consistent with earlier observations regarding higher risk groups in the literature.^1^ It is unclear if the lack of representation of patients from the developing world is either due to a lower detection rate of actual cases or if LyE is simply more prevalent in the industrialized world, similar to trends of other autoimmune gastrointestinal diseases such as inflammatory bowel disease.^16^ None of the patients in our study were younger than 18 years old since there was no treatment data for pediatric LyE at the time of our search; this suggests a lack of studies in this area. The complete lack of pediatric data draws attention to an important gap in knowledge where further investigation is needed.

The clinical presentation of patients was broad. The most common symptoms were dysphagia and heartburn, which makes it easy to confuse LyE with more common disease entities such as GERD or EoE.^17^^,^^18^ In clinical practice, many patients with heartburn are empirically treated with acid suppression, which explains the sizeable number of patients in our study who were already receiving PPIs prior to diagnosis with LyE.^19^ The observation that several patients also had features of GERD implies these conditions may coexist, rather than being mutually exclusive. Additionally, a lack of symptoms also does not rule out LyE, given that 8 patients were asymptomatic at the time of diagnosis.

LyE was frequently associated with endoscopic abnormalities, including esophageal rings, strictures, and exudates.^3^ However, close to 30% (46 patients) of patients exhibited normal endoscopic findings, underscoring the critical role of histologic confirmation through biopsy. While rare, reports of spontaneous esophageal perforation suggest the potential for serious complications, highlighting the importance of vigilant clinical monitoring.

Most of the studies analyzed in this review aimed to treat LyE in a manner similar to EoE, mainly using PPIs or topical steroids, although no elimination diets were attempted. We found that more patients had a symptomatic, endoscopic, and histologic response to steroid monotherapy (topical or systemic) as an initial treatment compared to PPI monotherapy, although patients on steroid monotherapy had a higher symptomatic recurrence rate. For patients whose symptoms did not respond to a PPI as an initial treatment, topical steroids were effective in 1/3 patients. The higher response rate to steroids may be a result of the immunosuppressive effects of the drug that directly inhibits inflammatory mechanisms in LyE, in contrast to the acid-suppressing effects of PPIs. This also makes sense in the context of previous evidence suggesting that LyE is not likely to be related to GERD.^20^^,^^21^ There was a high proportion of patients who responded well to biologics too, lending further support to the hypothesis that LyE is likely a primarily autoimmune process in nature. Interestingly, PPI and steroid combination therapy did not produce a higher response rate compared to steroid monotherapy. Endoscopic dilation was helpful in symptomatic relief of esophageal strictures either at the time of initial therapy or as a second-line treatment, but did not contribute to histologic resolution. Unusually, the patient from Lee *et al*.^12^ had self-resolution of symptoms but not histologic resolution without any treatment, potentially suggesting histology, and symptomatology may not always be directly correlated.

Our findings above were very similar to patterns previously reported for the response to treatment of EoE. Patients with EoE had a higher rate of response to topical steroids compared to PPI.^22^ Additionally, there was a high rate of symptomatic recurrence to either type of medication.^23^ Finally, patients with EoE who do not respond to initial treatment with PPIs can be switched to topical steroids for an improved response.^24^ This raises the possibility that LyE and EoE may share mechanistic similarities, although direct comparative studies remain lacking.

**Fig. 2:**
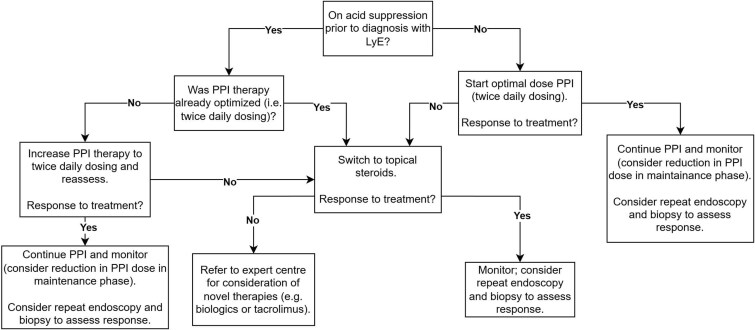
Proposed treatment algorithm for LyE.

Side effects of swallowed steroids include esophageal candidiasis, while side effects of PPIs consist of increased risk of infections such as *C. difficile* and nutrient malabsorption.^25^^,^^26^ Given the generally more benign side effect profile of PPIs in contrast to steroids, it would be reasonable to trial a PPI as an initial therapy in patients with LyE who have not been on optimal acid suppression previously, followed by a switch to topical steroids such as budesonide or fluticasone should PPI therapy remain ineffective. If topical steroids are insufficient to relieve symptoms, patients should be considered for repeat upper endoscopy and possible balloon dilation of any strictures. On a case-by-case basis subject to specialist consultation, biologics or other immunosuppressive drugs may be used to treat severe refractory cases of LyE. We therefore propose the following treatment algorithm below (Fig.  2).

There were several limitations to our review. Much of the data was derived from case reports and small retrospective series, often lacking standardized treatment protocols or consistent follow-up. For many studies, the time to clinical response or recurrence was not reported. Our intention was to evaluate treatment efficacy using standardized criteria, with symptomatic response defined as improvement or resolution of dysphagia or chest discomfort, endoscopic response defined as resolution or reduction of mucosal abnormalities (e.g. rings, furrows, exudates, or strictures), and histologic response defined as reduction or normalization in intraepithelial lymphocyte counts with resolution of associated spongiosis. However, there was a lack of specific definitions for treatment response in most primary studies. We therefore relied on the best possible information available to maximize the utility of the available data. Given the small sample sizes of patients receiving treatment, inability to calculate odds ratios based on the data available, and the heterogenous outcome reporting (i.e. inability to pool odds ratios for each outcome) of the studies analyzed, we were limited to only descriptive statistics and unable to formally analyze the comparative effectiveness of different treatment modalities via a meta-analysis. Additionally, all the studies in our review (case reports and retrospective series) were vulnerable to publication bias and confounding inherent to the design of these studies, with no control subjects included. Many of the included primary studies in this systematic review did not consistently report all of symptomatic response, endoscopic response, or histologic response for many of the examined treatment modalities, leaving our results vulnerable to reporting bias. Finally, the average follow-up duration of 8.98 months was relatively short, which would be a limitation in assessment of recurrence rates and longer-term efficacy.

In conclusion, while a range of therapeutic interventions have been used in LyE, treatment remains largely empirical, the most common of which are PPIs and topical steroids. There is a need for further prospective research such as a randomized control trial to formally compare the efficacies of the various therapeutic options for LyE, and to better define optimal treatment strategies and understand the disease’s natural course.

## Supplementary Material

APPENDIX_A_Search_strategy_reference_list_doaf112

APPENDIX_B_study_characteristics_doaf112

APPENDIX_C_quality_assessment_doaf112

## Notes

Specific author contributions: Conception and design of this work was conducted by BN and FJ. BN and CFC performed study screening and data extraction. BN and CFC performed statistical analysis of the data. All authors (BN, CFC, FJ) contributed to drafting the manuscript, critical revisions, and provided final approval for publication. FJ is the guarantor of this manuscript.

Financial support: This was an unfunded study.

Data availability: The data underlying this study have all been included either as part of the main manuscript or its appendices.

Ethical considerations: No research ethics board review was required as this study only relied on publicly published data, per University of British Columbia Ethics Board guidelines. This study was performed in accordance with the principles of the Declaration of Helsinski.
