# Supplementary material for: A systematic review of therapeutic options for lymphocytic esophagitis
Source: Dis Esophagus. 2025 Dec 5;38(6):doaf112. doi: 10.1093/dote/doaf112 (PMC12680012; doi:10.1093/dote/doaf112)
Supplement: APPENDIX_A_Search_strategy_reference_list_doaf112 [file appendix_a_search_strategy_reference_list_doaf112.docx]

**APPENDIX A: Search Strategy and Included Studies Reference List**

**MEDLINE <1946 – 2025> Search Strategy**

1. ("lymphocytic esophagitis" or "lymphocyte-predominant esophagitis").mp. [mp=title, book title, abstract, original title, name of substance word, subject heading word, floating sub-heading word, keyword heading word, organism supplementary concept word, protocol supplementary concept word, rare disease supplementary concept word, unique identifier, synonyms, population supplementary concept word, anatomy supplementary concept word]

2. exp Proton Pump Inhibitors/

3. ("proton pump inhibitor*" or omeprazole).mp. [mp=title, book title, abstract, original title, name of substance word, subject heading word, floating sub-heading word, keyword heading word, organism supplementary concept word, protocol supplementary concept word, rare disease supplementary concept word, unique identifier, synonyms, population supplementary concept word, anatomy supplementary concept word]

4. 2 or 3

5. 1 and 4

6. (balloon or "endoscopic dilation").mp. [mp=title, book title, abstract, original title, name of substance word, subject heading word, floating sub-heading word, keyword heading word, organism supplementary concept word, protocol supplementary concept word, rare disease supplementary concept word, unique identifier, synonyms, population supplementary concept word, anatomy supplementary concept word]

7. exp Budesonide/

8. Budesonide.mp. [mp=title, book title, abstract, original title, name of substance word, subject heading word, floating sub-heading word, keyword heading word, organism supplementary concept word, protocol supplementary concept word, rare disease supplementary concept word, unique identifier, synonyms, population supplementary concept word, anatomy supplementary concept word]

9. 7 or 8

10. 4 or 6 or 9

11. 1 and 10

12. exp Steroids/

13. steroid*.mp. [mp=title, book title, abstract, original title, name of substance word, subject heading word, floating sub-heading word, keyword heading word, organism supplementary concept word, protocol supplementary concept word, rare disease supplementary concept word, unique identifier, synonyms, population supplementary concept word, anatomy supplementary concept word]

14. 12 or 13

15. Fluticasone.mp. [mp=title, book title, abstract, original title, name of substance word, subject heading word, floating sub-heading word, keyword heading word, organism supplementary concept word, protocol supplementary concept word, rare disease supplementary concept word, unique identifier, synonyms, population supplementary concept word, anatomy supplementary concept word]

16. Prednisone.mp. [mp=title, book title, abstract, original title, name of substance word, subject heading word, floating sub-heading word, keyword heading word, organism supplementary concept word, protocol supplementary concept word, rare disease supplementary concept word, unique identifier, synonyms, population supplementary concept word, anatomy supplementary concept word] 58,568

17. 15 or 16

18. Pantoprazole.mp. [mp=title, book title, abstract, original title, name of substance word, subject heading word, floating sub-heading word, keyword heading word, organism supplementary concept word, protocol supplementary concept word, rare disease supplementary concept word, unique identifier, synonyms, population supplementary concept word, anatomy supplementary concept word]

19. Rabeprazole.mp. [mp=title, book title, abstract, original title, name of substance word, subject heading word, floating sub-heading word, keyword heading word, organism supplementary concept word, protocol supplementary concept word, rare disease supplementary concept word, unique identifier, synonyms, population supplementary concept word, anatomy supplementary concept word]

20. Esomeprazole.mp. [mp=title, book title, abstract, original title, name of substance word, subject heading word, floating sub-heading word, keyword heading word, organism supplementary concept word, protocol supplementary concept word, rare disease supplementary concept word, unique identifier, synonyms, population supplementary concept word, anatomy supplementary concept word]

21. Dexlansoprazole.mp. [mp=title, book title, abstract, original title, name of substance word, subject heading word, floating sub-heading word, keyword heading word, organism supplementary concept word, protocol supplementary concept word, rare disease supplementary concept word, unique identifier, synonyms, population supplementary concept word, anatomy supplementary concept word]

22. 18 or 19 or 20 or 21

23. Elimination diet.mp. [mp=title, book title, abstract, original title, name of substance word, subject heading word, floating sub-heading word, keyword heading word, organism supplementary concept word, protocol supplementary concept word, rare disease supplementary concept word, unique identifier, synonyms, population supplementary concept word, anatomy supplementary concept word]

24. Six food elimination diet.mp. [mp=title, book title, abstract, original title, name of substance word, subject heading word, floating sub-heading word, keyword heading word, organism supplementary concept word, protocol supplementary concept word, rare disease supplementary concept word, unique identifier, synonyms, population supplementary concept word, anatomy supplementary concept word]

25. 23 or 24

26. Allergy testing.mp. [mp=title, book title, abstract, original title, name of substance word, subject heading word, floating sub-heading word, keyword heading word, organism supplementary concept word, protocol supplementary concept word, rare disease supplementary concept word, unique identifier, synonyms, population supplementary concept word, anatomy supplementary concept word]

27. Savary dilation.mp. [mp=title, book title, abstract, original title, name of substance word, subject heading word, floating sub-heading word, keyword heading word, organism supplementary concept word, protocol supplementary concept word, rare disease supplementary concept word, unique identifier, synonyms, population supplementary concept word, anatomy supplementary concept word]

28. Biologic.mp. [mp=title, book title, abstract, original title, name of substance word, subject heading word, floating sub-heading word, keyword heading word, organism supplementary concept word, protocol supplementary concept word, rare disease supplementary concept word, unique identifier, synonyms, population supplementary concept word, anatomy supplementary concept word]

29. Anti-TNF inhibitor.mp. [mp=title, book title, abstract, original title, name of substance word, subject heading word, floating sub-heading word, keyword heading word, organism supplementary concept word, protocol supplementary concept word, rare disease supplementary concept word, unique identifier, synonyms, population supplementary concept word, anatomy supplementary concept word]

30. 28 or 29

31. Conservative management.mp. [mp=title, book title, abstract, original title, name of substance word, subject heading word, floating sub-heading word, keyword heading word, organism supplementary concept word, protocol supplementary concept word, rare disease supplementary concept word, unique identifier, synonyms, population supplementary concept word, anatomy supplementary concept word]

32. 17 or 22 or 25 or 26 or 27 or 30 or 31

33. 4 or 6 or 9 or 14 or 32

34. 1 and 33

**Embase <1974 – 2025> Search Strategy**

1. exp proton pump inhibitor/ or exp lansoprazole/ or exp linaprazan/

2, ("lymphocytic esophagitis" or "lymphocyte-predominant esophagitis").mp. [mp=title, abstract, heading word, drug trade name, original title, device manufacturer, drug manufacturer, device trade name, keyword heading word, floating subheading word, candidate term word]

3. ("proton pump inhibitor*" or omeprazole).mp. [mp=title, abstract, heading word, drug trade name, original title, device manufacturer, drug manufacturer, device trade name, keyword heading word, floating subheading word, candidate term word]

4. 1 or 3

5. (balloon or "endoscopic dilation").mp. [mp=title, abstract, heading word, drug trade name, original title, device manufacturer, drug manufacturer, device trade name, keyword heading word, floating subheading word, candidate term word]

6. balloon dilatation/

7. 5 or 6

8. budesonide/

9. Budesonide.mp.

10. 8 or 9

11. exp steroid/ or exp steroid hormone/

12. steroid*.mp. [mp=title, abstract, heading word, drug trade name, original title, device manufacturer, drug manufacturer, device trade name, keyword heading word, floating subheading word, candidate term word]

13. 11 or 12

14. 4 or 7 or 10 or 13

15. 2 and 14

**Google Scholar Search Strategy**

Lymphocytic esophagitis treatment

**Reference List of Included Studies**

Amin S, Munankami S, Desai P, Altomare J, Shah N. Immune Checkpoint Inhibitor-Induced Lymphocytic Esophagitis. *Cureus.* 2023;15(6):e39920. doi: 10.7759/cureus.39920.

Aboona MB, Yost KK, Aguon PM, Fung BM, Nanda R. Lymphocytic Esophagitis Presenting With Food Impaction. *Cureus*. 2023;15(8):e42873. doi: 10.7759/cureus.42873.

Beales ILP. Successful Treatment of Refractory Lymphocytic Esophagitis With Vedolizumab. *Am J Gastroenterol*. 2019;114(9):1555–6. doi: 10.14309/ajg.0000000000000350.

Che C, Dworkin B. Lymphocytic Esophagitis in Crohnʼs Disease. *Am J Gastroenterol*. 2013;108:S199.

Atiq I, Amin N, Usama Sakhawat, Ramar G, Khan A, Chowdhury N. S2344 Lymphocytic Esophagitis: A Rare and Rising Cause of Dysphagia. *Am J Gastroenterol*. 2022;117(10S):e1575–6. doi: 10.14309/01.ajg.0000866016.28778.60

Shipley LC, Al Momani LA, Locke A, Young M. Lymphocytic Esophagitis: A Rare Disease on the Rise. *Cureus*. 2018;10(2):e2153. doi: 10.7759/cureus.2153

Alsamman Y, Wan DW. Lymphocytic Esophagitis and Dysphagia: Cause or Association? *Am J Gastroenterol*. 2015;110:S252–2.

Bayoumi M, Ghai MB, Yost KK, Robertson WA, Telken D, Drewitz D. S2342 Lymphocytic Esophagitis: A Rare Etiology for Persistent Heartburn. *Am J Gastroenterol*. 2022;117(10S):e1575–5. doi: 10.14309/01.ajg.0000866008.29380.23

Becheanu G, Doina Istratescu, Preda CM, Mircea Manuc, Mircea Mihai Diculescu, Gheorghe C. Lymphocytic Esophagitis Successfully Treated by Esophageal Balloon Dilation and Topical Budesonide. *J Gastrointestin Liver Dis*. 2019;28(4):379–9. doi: 10.15403/jgld-217.

Farooqui Z, Shine A, Rogers B. S2054 A Difficult Case of Lymphocytic Esophagitis. *Am J Gastroenterol.* 2021;116(1):S889–90. doi: 10.14309/01.ajg.0000781748.10058.9d

Figueiredo PC, Pinto-Marques P, Borralho P, Freitas J. Unusual Cause for Smoldering Dysphagia. *Dysphagia*. 2013;29(2):283–5. doi: 10.1007/s00455-013-9489-2.

Havre RF, Trine Hallager, Evangelos Kalaitzakis. An 87-Year-Old Woman With Recurrent Dysphagia. *Gastroenterology*. 2016;151(6):1085–6. doi: 10.1053/j.gastro.2016.07.049.

Hendy PJ, Florin TH. Spontaneous oesophageal perforation: an unreported complication of lymphocytic oesophagitis. *Gut*. 2013;62(11):1668–9. doi: 10.1136/gutjnl-2013-305455.

Islam S, Lee A, Lampe G. Lymphocytic esophagitis: An Australian (Queensland) case series of a newly recognized mimic of eosinophilic esophagitis. *JGH Open*. 2019;3(5):400–4. doi: 10.1002/jgh3.12175.

Jideh B, Keegan A, Weltman M. Lymphocytic esophagitis: Report of three cases and review of the literature. *World J Clin Cases*. 2016;4(12):413. doi: 10.12998/wjcc.v4.i12.413.

Kasirye Y, John A, Rall C, Resnick J. Lymphocytic Esophagitis Presenting as Chronic Dysphagia. *Clin Med Res*. 2012;10(2):83–4. doi: 10.3121/cmr.2011.1009.

Lavette LE, Young C, Gibbs A, Mills AM, Stelow EB, Copland AP. S2795 Topical Steroids: An Effective Treatment Option for Lymphocytic Esophagitis. *Am J Gastroenterol*. 2023;118(10S):S1904–4. 10.14309/01.ajg.0000960820.60772.de.

Leung D, Sirjie AM, Bao F, Tsai F, Coyle W, Nhu QM. S2255 Oral Viscous Budesonide Ameliorates PPI-Refractory Lymphocytic Esophagitis: A Longitudinal Clinical, Endoscopic, and Histologic Outcome Case Report. *Am J Gastroenterol*. 2022;117(10S):e1521–1. doi: 10.14309/01.ajg.0000865660.07542.c1.

Mandaliya R, DiMarino AJ, Cohen S. Lymphocytic esophagitis mimicking eosinophilic esophagitis. *Ann Gastroenterol*. 2012;25(4):355-7.

Meka KC, Farshadsefat S, Gamarra F, Maas LC. Lymphocytic Esophagitis (LyE)-Associated With a Lichenoid Esophagitis Pattern (LeP) Presenting With Dysphagia and Esophagogastric Junction Outflow Obstruction (EGJOO) on High-Resolution Esophageal Manometry (HRM). *Am J Gastroenterol.* 2017;112:S914.

Nieves K, Calo D, Tilara A, Mendelsohn R. An unusual cause of dysphagia. *Am J Gastroenterol*. 2013;108:S197-8.

Paparoupa M, Linnemüller S, Schuppert F. Off-label use of Budesonide suspensions to treat a patient with lymphocytic esophagitis. *Laryngorhinootologie.* 2019 Oct;98(10):718-721. doi: 10.1055/a-0747-7023.

Paramsothy J, Villa N, Obanor S, Iswara K, Pittman ME, Zivari K. S2739 Lymphocytic Esophagitis in a Patient With Progressive Dysphagia and Rapid Weight Loss. *Am J Gastroenterol*. 2023;118:S1872–2. doi: 10.14309/01.ajg.0000960596.77984.79.

Prevallet A, Leung D, Khanna R, Chandradas S. S2126 An Atypical Case of Lymphocytic Esophagitis in a Young Female. *Am J Gastroenterol*. 2021;116(1):S915–5. doi: 10.14309/01.ajg.0000782036.45528.34.

Reddy S, Feller E, Wang LJ, Shah S. Lymphocytic Esophagitis in Crohn’s Disease: Understanding the Significance. *Am J Gastroenterol*. 2014;109:S236

Singhal P, Nguyen SH. S3547 Lymphocytic Esophagitis: An Unlikely Mimicker of Gastroesophageal Reflux Disease in the COVID-19 Era. *Am J Gastroenterol*. 2021;116(1):S1456–6. doi: 10.14309/01.ajg.0000787720.56748.5d.

Sloan J, Sandhu N, Miick R, Govil Y. Proton Pump Inhibitor-Induced Remission of Lymphocytic Esophagitis. *ACG Case Rep J.* 2016;3(1):e195–5. doi: 10.14309/crj.2016.168.

Hesham Tayel, Kim B, Martinez-Tapia A, Hussam Tayel, Duarte-Chavez R, Matin A. Lymphocytic Esophagitis Masquerading as Gastroesophageal Reflux Disease-Induced Peptic Strictures. *Am J Gastroenterol.* 2018;113:S1040–0.

Townsend AS, Rizk K, Clayton S. A Case of PPI Responsive Lymphocytic Esophagitis. *Am J Gastroenterol*. 2016;111:S800.

Wojas O, Żalikowska-Gardocka M, Krzych-Fałta E *et al*. A case of lymphocytic esophagitis in a woman with multiple allergies. *Allergy Asthma and Clin Immunol*. 2021;17:56. doi: 10.1186/s13223-021-00558-x.

Young CL, Doran A. S2036 Lymphocytic Esophagitis: A Rare Etiology of Esophageal Tear. *Am J Gastroenterol.* 2021;116(1):S883–3. doi: 10.14309/01.ajg.0000781676.20262.4e.

Zhang Z, Jain D, Brand M. Ringed Esophagus Secondary to Lymphocytic Esophagitis. *Gastroenterol Hepatol* (N Y). 2016;12(4):237–9.

Jacob AM, Rao S, Sun, Borum ML, Schueler SA. S417 Lymphocytic Esophagitis: Assessment of the Variable Clinical Presentation. *Am J Gastroenterol*. 2021;116(1):S185–5. doi: 10.14309/01.ajg.0000774140.76216.70.

Lagrotteria A, Collins AW, Someili A, Narula N. A141 Spontaneous esophageal perforation: an unreported complication of lymphocytic oesophagitis*. J Can Assoc Gastroenterol*. 2021;4(Supplement_1):128–9. doi: 10.1093/jcag/gwab002.139.

Maejima R, Uno K, Iijima K, Fujishima F, Noguchi T, Ara N, Asano N, Koike T, Imatani A, Shimosegawa T. A Japanese case of lymphocytic esophagitis. *Dig Endosc*. 2015 Nov;28(4):476–80. doi: 10.1111/den.12578.

Schoepfer AM, Asikainen S, Biedermann L, Kreienbühl A, Godat A, Straumann A, Greuter T. Swallowed Topical Tacrolimus Induces Clinical and Histological Remission in a Subset of Patients with Severe Lymphocytic Esophagitis. *Inflamm Intest Dis*. 2025 Jan 21;10(1):41–9. doi: 10.1159/000542812.

Lee ME, Ghosh G, Yantiss R, Katz PO. Lymphocyte-Predominant Esophagitis Rarely Improves With Proton Pump Inhibitor Therapy. *Foregut*. 2022;2(2):212–6. doi: 10.1177/26345161221108688

Pizzuti J, Wander P, Lech D, Ashamalla M, Rishi A, Lee C. 1806 When the Cure Is the Cause: Checkpoint Inhibitor-Induced Lymphocytic Esophagitis. *Am J Gastroenterol*. 2019;114(1):S1014–4. doi: 10.14309/01.ajg.0000596756.20000.cb.
