# Supplementary material for: A systematic review of therapeutic options for lymphocytic esophagitis
Source: Dis Esophagus. 2025 Dec 5;38(6):doaf112. doi: 10.1093/dote/doaf112 (PMC12680012; doi:10.1093/dote/doaf112)
Supplement: APPENDIX_B_study_characteristics_doaf112 [file appendix_b_study_characteristics_doaf112.docx]

**APPENDIX B: Study Characteristics**

**Table B.1: Study Characteristics and Patient Characteristics**

| **Reference** | **Location** | **Study Design** | **Demographics: Sex (M/F) and Age (years)** | **Past Medical History** | **Previous Medications** |
| --- | --- | --- | --- | --- | --- |
| **Amin et al. 2023** | USA | Case Study | M: n=1  Age: 79 | RCC | Nivolumab |
| **Aboona et al. 2023** | USA | Case Study | M: n=1  Age: 71 | HTN, tobacco and alcohol use | N/A |
| **Beales et al. 2019** | UK | Case Study | M: n=1  Age: 29 | None | None |
| **Che et al. 2013** | USA | Case Study | F: n=1  Age: 37 | CD | N/A |
| **Atiq et al. 2022** | USA | Case Study | M: n=1  Age: 83 | CAD, HTN, emphysema, GERD, and alcohol use | PPI |
| **Shipley et al. 2018** | USA | Case Study | M: n=1  Age: 71 | SCC tongue, GERD, HTN | PPI |
| **Alsamman et al. 2015** | USA | Case Study | M: n=1  Age: 87 | Barrett’s esophagus, CE, tubular adenoma of colon, COPD, HTN, right carotid endarterectomy, CKD | N/A |
| **Bayoumi et al. 2022** | USA | Case Study | F: n=1  Age: 24 | Ulcerative colitis, tobacco use | Omeprazole, infliximab |
| **Becheaunu et al. 2019** | Romania | Case Study | F: n=1  Age: 59 | None | None |
| **Farooqui et al. 2021** | USA | Case Study | F: n=1  Age: 81 | None | N/A |
| **Figuieredo et al. 2014** | Portugal | Case Study | F: n=1  Age: 30 | Behcet disease, SLE | Colchicine, hydroxychloroquine, sucralfate |
| **Lee et al. 2022** | USA | Retrospective Case Series | M: n=17  F: n=45  Mean age: 55.8 | -CD: n=19  -Rheumatoid arthritis: n=12  -Autoimmune thyroid disease: n=10 | N/A |
| **Havre et al. 2016** | Denmark | Case Study |  | N/A | None |
| **Hendy et al. 2013** | Australia | Case Study |  | None | PPI, clindamycin, ciprofloxacin |
| **Islam et al. 2019** | Australia | Case Study |  | Alcohol and tobacco use, chronic pancreatitis, HTN | Fenofibrate, omeprazole, irbesartan |
| **Jacob et al. 2021** | USA | Retrospective Case Series | M: n=11  F: n=7  Mean age: 55.2 | N/A | Acid suppressing medications: n=3 |
| **Jideh et al. 2016** | Australia | Retrospective Case Series | M: n=1  F: n=2  Mean age: 65 | -None: n=1  -GERD and COPD: n=1  -IBS and hypothyroidism: n=1 | -None: n=1  -Corticosteroids and antibiotics: n=1  -N/A: n=1 |
| **Kasirye et al. 2012** | USA | Case Study | F: n=1  Age: 60 | Diabetes, CKD, LP | Lansoprazole, famotidine |
| **Lagrotteria et al. 2021** | Canada | Case Study | M: n=1  Age: 31 | None | PPI, antibiotics |
| **Lavette et al. 2023** | USA | Retrospective Case Series | M: n=1  F: n=3  Mean age: 38.5 | -Gastroparesis and gastric bypass: n=1  -Prior esophageal tear: n=1  -None: n=1  -Alcohol and tobacco use: n=1 | -N/A: n=3  -None: n=1 |
| **Leung et al. 2022** | USA | Case Study | F: n=1  Age: 77 | Buccal LP, lymphocytic colitis, depression, anxiety, osteoarthritis | Omeprazole |
| **Maejima et al. 2016** | Japan | Case Study | M: n=1  Age: 68 | None | None |
| **Mandaliya et al. 2012** | USA | Case Study | M: n=1  Age: 74 | Lymphoma, ES | N/A |
| **Meka et al. 2017** | USA | Case Study | F: n=1  Age: 58 | N/A | PPI |
| **Nieves et al. 2013** | USA | Case Study | M: n=1  Age: 45 | Hodgkin lymphoma | PPI |
| **Paparoupa et al. 2019** | Germany | Case Study | M: n=1  Age: 62 | N/A | None |
| **Paramsothy et al. 2023** | USA | Case Study | F: n=1  Age: 65 | N/A | N/A |
| **Pizzuti et al. 2019** | USA | Case Study | F: n=1  Age: 74 | RCC | Nivolumab |
| **Prevallet et al. 2021** | USA | Case Study | F: n=1  Age: 25 | N/A | N/A |
| **Reddy et al. 2014** | USA | Case Study | M: n=1  Age: 49 | CD, arthritis, GERD, tobacco use | Adalimumab, omeprazole |
| **Schoepfer et al. 2024** | Switzerland | Retrospective Case Series | M: n=4  F: n=3  Mean age: 71.3 | -GERD: n=1 | PPI: n=1 |
| **Singhal et al. 2021** | USA | Case Study | M: n=1  Age: 60 | GERD, COVID-19 | N/A |
| **Sloan et al. 2016** | USA | Case Study | M: n=1  Age: 38 | Epilepsy | Phenytoin |
| **Tayel et al. 2018** | USA | Case Study | M: n=1  Age: 80 | Tobacco use, GERD, ES, CE | N/A |
| **Townsend et al. 2016** | USA | Case Study | M: n=1  Age: 53 | Pulmonary nodule, tobacco and alcohol use | None |
| **Wojas et al. 2021** | Poland | Case Study | F: n=1  Age: 28 | Asthma, contact dermatitis, obesity | N/A |
| **Yost et al. 2022** | USA | Case Study | M: n=1  Age: 71 | HTN, tobacco and alcohol use | N/A |
| **Young et al. 2021** | USA | Case Study | F: n=1  Age: 35 | None | PPI |
| **Zhang et al. 2016** | USA | Case Study | F: n=1  Age: 66 | Bipolar disorder, opioid overdose, cholecystectomy, tobacco use | Clonazepam, ziprasidone, buprenorphine, naloxone |
| - M: male; F: female - RCC: renal cell carcinoma; HTN: hypertension; CD: Crohn’s disease; CAD: coronary artery disease; GERD: gastroesophageal reflux disorder; SCC: squamous cell carcinoma; CE: candida esophagitis; ES: esophageal stricture; CKD: chronic kidney disease, COPD: chronic obstructive pulmonary disease; LP: lichen planus; IBS: irritable bowel syndrome - PPI: proton pump inhibitor - N/A: no data | | | | | |

**Table B.2: Lymphocytic Esophagitis Diagnostic Features**

| **Reference** | **Presenting Symptoms** | **Endoscopic Findings** | **Pathology** |
| --- | --- | --- | --- |
| **Amin et al. 2023** | Dysphagia, weight loss, abdominal pain | Schatzki’s ring, esophageal inflammation, furrows | Lymphocytic infiltration of the epithelium, dyskeratotic keratinocytes, and acanthosis |
| **Aboona et al. 2023** | Dysphagia, food impaction | Food bolus, esophageal ulcers, esophageal stricture | Squamous mucosa with features of lymphocytic esophagitis |
| **Beales et al. 2019** | Dysphagia, heartburn, food bolus obstruction | Normal | Hyperplastic non-keratinized squamous epithelium with spongiosis and notable intraepithelial lymphocytosis with a prominent peripapillary distribution (65–120 lymphocytes/hpf), without granulocytic or eosinophilic infiltration |
| **Che et al. 2013** | Dysphagia, odynophagia, abdominal pain | Esophageal stenosis, rings | Basal cell hyperplasia, extension of papillae, and marked lymphocytic exocytosis. Immunoperoxidase stains were positive for CD3 T cells and the lymphocyte count was 100/hpf |
| **Atiq et al. 2022** | Dysphagia, generalized weakness, odynophagia, heartburn, weight loss | Normal | Squamous mucosa with increased intraepithelial and patchy peripapillary lymphocytes, along with reactive epithelial changes |
| **Shipley et al. 2018** | Dysphagia | Esophageal stenosis, white exudates | Peripapillary intraepithelial lymphocytes |
| **Alsamman et al. 2015** | Dysphagia | Esophageal rings, Schatzki ring | Consistent with lymphocytic esophagitis |
| **Bayoumi et al. 2022** | Heartburn, epigastric discomfort | Mucosal hyperemia, furrows, white exudates | Dense infiltrates of intraepithelial lymphocytes and spongiosis |
| **Becheaunu et al. 2019** | Dysphagia, weight loss | Esophageal stenosis | Intraepithelial lymphocytosis, peripapillary spongiosis, with many squiggle cells |
| **Farooqui et al. 2021** | Recurrent dysphagia, poor oral intake, weight loss | Stenosis, mucosal inflammation, exudate | Lymphocytic infiltration at both proximal and distal esophagus, and frequent dyskeratotic cells without eosinophilia or granulocytosis |
| **Figuieredo et al. 2014** | Heartburn, odynophagia | Mucosal irregularities | Marked acanthosis and papillomatosis of the squamous epithelium; intense lymphoplasmacytic infiltrate with an increased number of intraepithelial lymphocytes |
| **Lee et al. 2022** | -Dysphagia: n=36  -Heartburn: n=12  -Abdominal pain: n=11  -Asymptomatic: n=8 | -Normal: n=14  -Edema: n=8  -Rings: n=18  -Exudate: n=9  -Furrows: n=5  -Stricture: n=7  -Esophagitis: n=20  -Findings resembling EoE: n=26 | Panesophageal lymphocyte distribution |
| **Havre et al. 2016** | Dysphagia | Esophageal stricture, rings | Dense intraepithelial lymphocytic infiltration of the peripapillary fields without neutrophilic or eosinophilic granulocytes, spongiosis |
| **Hendy et al. 2013** | Odynophagia, severe chest pain, fever >40 degrees, shock | Normal | Large numbers of intramucosal lymphocytes, clustered focally at peripapillary zones, with associated spongiosis; no eosinophilia |
| **Islam et al. 2019** | Progressive dysphagia, odynophagia, weight loss | Reddened mucosa, white exudates | Extensive peripapillary intraepithelial infiltration with lymphocytes; >50-80 lymphocytes per hpf |
| **Jacob et al. 2021** | -Heartburn: n=13  -Dysphagia: n=12  -Odynophagia: n=5/18  -Weight loss: n=4  -N/V: n=7 | -Normal: n=4  -Plaques: n=4  -Rings: n=4  -La class A esophagitis: n=3  -Abnormal appearance: n=14 | N/A |
| **Jideh et al. 2016** | -Dysphagia and food bolus impaction: n=1  -Dysphagia: n=1  -Dysphagia, globus sensation: n=1 | -Normal: n=2  -Linear furrows, stricture: n=1 | -Heavy lymphocytic infiltrate with occasional neutrophils and eosinophils (n=1)  -Prominent basal cell hyperplasia with intercellular edema and a heavy intra-epithelial lymphocytic infiltrate (up to 60/hpf) in a peripapillary distribution without granulocytes (n=1)  -Significant intraepithelial lymphocytic infiltrate and minimal eosinophils or neutrophils (n=1) |
| **Kasirye et al. 2012** | Dysphagia, heartburn | Rings, furrows | Intraepithelial lymphocytosis in a peripapillary distribution |
| **Lagrotteria et al. 2021** | Food bolus impaction, odynophagia, chest discomfort | Mucosal tear | Marked infiltration of intraepithelial lymphocytes |
| **Lavette et al. 2023** | -Dysphagia: n=4 | -Mucosal changes: n=1  -Normal: n=1  -N/A: n=1  -Rings with stenosis: n=1 | -Numerous intraepithelial lymphocytes and spongiosis (n=4) |
| **Leung et al. 2022** | Dysphagia | Esophageal stenosis, diffuse pallor, edema, decreased vascularity, diffuse rings, mucosal scarring, strictures | >25 intraepithelial lymphocytes (IEL)/hpf proximally and distally |
| **Maejima et al. 2016** | Dysphagia, heartburn | White exudate, pale mucosa, furrows, stricture | Infiltration of interepithelial lymphocytes primarily gathered in a peri-papillary distribution |
| **Mandaliya et al. 2012** | Dysphagia, food bolus impaction | Stricture, rings | Marked infiltration of exclusive intraepithelial lymphocytes in a peripapillary distribution |
| **Meka et al. 2017** | Dysphagia | Normal | LyE-consistent pathology |
| **Nieves et al. 2013** | Dysphagia | Normal | Increased number of intraepithelial lymphocytes and rare granulocytes predominantly in the peripapillary fields |
| **Paparoupa et al. 2019** | Dysphagia | Rings, furrows | >100 lymphocytes per hpf in peripapillary distribution |
| **Paramsothy et al. 2023** | Dysphagia, weight loss | Stricture | Intraepithelial lymphocytosis and edema |
| **Pizzuti et al. 2019** | Dysphagia, throat pain | Circumferential erythema, exudate | Dyskeratotic keratinocytes and lymphoplasmacytic infiltrate |
| **Prevallet et al. 2021** | Dysphagia | Schatzki ring | 30-35 lymphocytes/hpf |
| **Reddy et al. 2014** | Persistent N/V and diarrhea | Normal | Confirmed LyE pathology |
| **Schoepfer et al. 2024** | -Dysphagia: n=7 | N/A | N/A |
| **Singhal et al. 2021** | Reflux, regurgitation | Furrows, white plaques | Lymphocytosis coupled with decreased neutrophil and eosinophil counts |
| **Sloan et al. 2016** | Dysphagia, inability to swallow | Normal | Marked lymphocytic intraepithelial and lamina propria inflammation |
| **Tayel et al. 2018** | Dysphagia | White plaques, strictures | Confirmed LyE pathology |
| **Townsend et al. 2016** | None | Esophageal nodules | 100 lymphocytes/hpf |
| **Wojas et al. 2021** | Dysphagia, foreign body sensation, heartburn | Normal | >60 lymphocytes per hpf, acanthosis |
| **Yost et al. 2022** | Dysphagia | Ulcers, stricture | >20 intraepithelial lymphocytes per hpf with rare neutrophils and rare eosinophils with associated patchy, peripapillary lymphocytic infiltrate |
| **Young et al. 2021** | Acute chest pain and vomiting | Mucosal tear | Intraepithelial lymphocytes, and little intraepithelial granulocytes |
| **Zhang et al. 2016** | Dysphagia, epigastric discomfort | Esophageal rings | Mild basal cell hyperplasia and increased intraepithelial lymphocytes without any accompanying neutrophils and eosinophils |
| N/V: nausea/vomiting; EoE: eosinophilic esophagitis  N/A: no data  hpf: high-powered field; LyE: lymphocytic esophagitis | | | |

**Table B.3: Initial Treatment and Outcome Details**

| **Reference** | **Treatment** | **SR** | **ER** | **HR** | **Recurrence?** |
| --- | --- | --- | --- | --- | --- |
| **Amin et al. 2023** | Pantoprazole monotherapy | No | N/A | N/A | N/A |
| **Aboona et al. 2023** | Omeprazole + balloon dilation | Yes | Yes | N/A | No |
| **Beales et al. 2019** | Omeprazole monotherapy | No | N/A | No | N/A |
| **Che et al. 2013** | PPI + prednisone | No | No | No | N/A |
| **Atiq et al. 2022** | PPI + balloon dilation | Yes | N/A | N/A | N/A |
| **Shipley et al. 2018** | Topical corticosteroids + dilation | Yes | N/A | N/A | N/A |
| **Alsamman et al. 2015** | No treatment | Yes | N/A | N/A | No |
| **Bayoumi et al. 2022** | Pantoprazole | No | No | No | N/A |
| **Becheaunu et al. 2019** | Topical budesonide + balloon dilation | Yes | Yes | N/A | No |
| **Farooqui et al. 2021** | PPI | No | N/A | N/A | N/A |
| **Figuieredo et al. 2014** | PPI | Yes | No | No | Yes |
| **Lee et al. 2022** | -PPI: n=21  -Topical fluticasone: n=6  -Balloon dilation: n=4  -Biologics: n=6  -No treatment: n=1 | -PPI: n=5  -Topical fluticasone: n=6  -Balloon dilation: n=3  -Biologics: n=5 | -PPI: n=1  -Steroids: n=3  -Balloon dilation: n=2  -Biologics: n=4 | -PPI: n=1  -Steroids: n=3  -Biologics: n=3  -No treatment: n=0 | N/A |
| **Havre et al. 2016** | Pantoprazole | N/A | N/A | N/A | N/A |
| **Hendy et al. 2013** | Topical fluticasone | Yes | N/A | N/A | Yes |
| **Islam et al. 2019** | Fluticasone + Rabeprazole | Yes | No | No | No |
| **Jacob et al. 2021** | PPI: n=15 | PPI: n=6 | N/A | N/A | N/A |
| **Jideh et al. 2016** | None | Yes | N/A | N/A | No |
|  | PPI+H2RA+steroid | No | N/A | N/A | N/A |
|  | PPI + balloon dilation | Yes | Yes | No | Yes |
| **Kasirye et al. 2012** | Fluticasone | Yes | N/A | N/A | No |
| **Lagrotteria et al. 2021** | PPI | Yes | N/A | N/A | Yes |
| **Lavette et al. 2023** | PPI + budesonide | Yes | Yes | Yes | N/A |
|  | PPI + budesonide | Yes | N/A | No | N/A |
|  | PPI + fluticasone | No | Yes | Yes | N/A |
|  | PPI + budesonide | Yes | No | No | N/A |
| **Leung et al. 2022** | Omeprazole + balloon dilation | No | No | N/A | N/A |
| **Maejima et al. 2016** | PPI | No | No | No | N/A |
| **Mandaliya et al. 2012** | Balloon dilation + esophageal botulinum injection | N/A | No | No | N/A |
| **Meka et al. 2017** | PPI + fluticasone | Yes | No | No | Yes |
| **Nieves et al. 2013** | PPI + fluticasone | Yes | N/A | N/A | Yes |
| **Paparoupa et al. 2019** | Topical budesonide | Yes | Yes | Yes | No |
| **Paramsothy et al. 2023** | PPI + balloon dilation | Yes | N/A | N/A | No |
| **Pizzuti et al. 2019** | IV methylprednisolone | Yes | N/A | N/A | Yes |
| **Prevallet et al. 2021** | PPI + balloon dilation | Yes | N/A | N/A | No |
| **Reddy et al. 2014** | PPI + fluticasone | No | N/A | N/A | N/A |
| **Schoepfer et al. 2024** | Tacrolimus: n=7 | Tacrolimus: n=4 | N/A | Tacrolimus: n=3 | N/A |
| **Singhal et al. 2021** | PPI increased dosing | Yes | N/A | N/A | No |
| **Sloan et al. 2016** | Pantoprazole | Yes | N/A | Yes | No |
| **Tayel et al. 2018** | PPI + balloon dilation | Yes | N/A | N/A | No |
| **Townsend et al. 2016** | Omeprazole | N/A | Yes | Yes | No |
| **Wojas et al. 2021** | Pantoprazole | No | N/A | N/A | N/A |
| **Yost et al. 2022** | PPI + balloon dilation | Yes | Yes | N/A | No |
| **Young et al. 2021** | PPI | Yes | Yes | Yes | No |
| **Zhang et al. 2016** | Omeprazole | Yes | N/A | N/A | N/A |
| PPI: proton pump inhibitor; H2RA: H2 receptor antagonist  N/A: non-applicable or no data  SR: symptomatic response; ER: endoscopic response; HR: histologic response | | | | | |

**Table B.4: Second-Line Treatment and Outcome Details**

| **Reference** | **Treatment** | **Symptomatic Response** | **Endoscopic Response** | **Histologic Response** | **Recurrence** |
| --- | --- | --- | --- | --- | --- |
| **Amin et al. 2023** | Prednisone | N/A | N/A | N/A | N/A |
| **Beales et al. 2019** | Budesonide | No | N/A | No | N/A |
| **Che et al. 2013** | Balloon dilation | Yes | N/A | No | N/A |
| **Bayoumi et al. 2022** | Famotidine | Yes | N/A | N/A | No |
| **Farooqui et al. 2021** | Topical steroids | No | N/A | N/A | N/A |
| **Figuieredo et al. 2014** | Fluticasone + balloon dilation | No | N/A | N/A | N/A |
| **Havre et al. 2016** | Balloon dilation | Yes | Yes | N/A | No |
| **Jideh et al. 2016** | PPI + balloon dilation: n=1 | Yes | N/A | No | Yes |
| **Leung et al. 2022** | Budesonide | Yes | Yes | Yes | No |
| **Maejima et al. 2016** | Balloon dilation | Yes | Yes | N/A | Yes |
| **Meka et al. 2017** | Botulinum injection to LES | Yes | No | No | Yes |
| **Pizzuti et al. 2019** | PPI + topical steroid | Yes | No | No | Yes |
| **Wojas et al. 2021** | PPI + famotidine + itopride HCl | No | N/A | N/A | N/A |
| PPI: proton pump inhibitor  N/A: no data or non-applicable | | | | | |

**Table B.5: Third-Line Treatment and Outcome Details**

| **Reference** | **Treatment** | **Symptomatic Response** | **Endoscopic Response** | **Histologic Response** | **Recurrence** |
| --- | --- | --- | --- | --- | --- |
| **Beales et al. 2019** | Vedolizumab | Yes | N/A | Yes | No |
| **Farooqui et al. 2021** | Balloon dilation | N/A | No | N/A | Yes |
| **Meka et al. 2017** | Topical fluticasone | N/A | N/A | N/A | N/A |
| **Pizzuti et al. 2019** | PPI + topical sucralfate | Yes | N/A | N/A | No |
| **Wojas et al. 2021** | IV pantoprazole | Yes | N/A | N/A | N/A |
| N/A: no data or non-applicable  PPI: proton pump inhibitor | | | | | |

**Table B.6: Completeness of Treatment Outcome Reporting**

| Reference | **Symptomatic Response** | **Endoscopic Response** | **Histologic Response** | **Recurrence** |
| --- | --- | --- | --- | --- |
| Proportion of studies reporting outcome for first-line treatment | 36/39 | 14/39 | 16/39 | 17/39 |
| Proportion of studies reporting outcome for second-line treatment | 12/13 | 5/13 | 6/13 | 7/13 |
| Proportion of studies reporting outcome for third-line treatment | 3/5 | 1/5 | 1/5 | 3/5 |
