# Supplementary material for: A systematic review of therapeutic options for lymphocytic esophagitis
Source: Dis Esophagus. 2025 Dec 5;38(6):doaf112. doi: 10.1093/dote/doaf112 (PMC12680012; doi:10.1093/dote/doaf112)
Supplement: APPENDIX_C_quality_assessment_doaf112 [file appendix_c_quality_assessment_doaf112.docx]

**APPENDIX C: Quality Assessment of Included Studies**

**Johanna Briggs Institute Critical Appraisal Checklist for Case Reports**

| **Questions** | **Amin et al. 2023** | **Aboona et al. 2023** | **Beales et al. 2019** | **Che et al. 2013** | **Atiq et al. 2022** | **Shipley et al. 2018** | **Alsamman et al. 2015** | **Bayoumi et al. 2022** |
| --- | --- | --- | --- | --- | --- | --- | --- | --- |
| **Were patient demographic characteristics clearly described?** | Yes | Yes | Yes | Yes | Yes | Yes | Yes | Yes |
| **Was the patient history clearly described and presented as a timeline?** | Yes | Yes | Yes | Yes | Yes | Yes | Yes | Yes |
| **Was the current clinical condition of the patient on presentation clearly described?** | Yes | Yes | Yes | Yes | Yes | Yes | Yes | Yes |
| **Were diagnostic tests or assessment methods and the results clearly described?** | Yes | Yes | Yes | Yes | Yes | Yes | Yes | Yes |
| **Was the intervention(s) or treatment procedure(s) clearly described?** | Yes | Yes | Yes | Yes | No | Yes | Yes | Yes |
| **Was the post-intervention clinical condition clearly described?** | No | Yes | Yes | Yes | Yes | Yes | No | Yes |
| **Were adverse events (harms) or unanticipated events identified and described?** | No | No | No | No | No | Yes | No | Yes |
| **Does the case report provide takeaway lessons?** | Yes | Yes | Yes | No | No | Yes | No | Yes |
| **Overall appraisal: Include?** | Yes | Yes | Yes | Yes | Yes | Yes | Yes | Yes |

| **Questions** | **Becheaunu et al. 2019** | **Farooqui et al. 2021** | **Figuieredo et al. 2014** | **Havre et al. 2016** | **Hendy et al. 2013** | **Islam et al. 2019** | **Kasirye et al. 2012** | **Lagrotteria et al. 2021** |
| --- | --- | --- | --- | --- | --- | --- | --- | --- |
| **Were patient demographic characteristics clearly described?** | Yes | Yes | Yes | Yes | Yes | Yes | Yes | Yes |
| **Was the patient history clearly described and presented as a timeline?** | Yes | No | Yes | No | No | Yes | Yes | Yes |
| **Was the current clinical condition of the patient on presentation clearly described?** | Yes | Yes | Yes | Yes | Yes | Yes | Yes | Yes |
| **Were diagnostic tests or assessment methods and the results clearly described?** | Yes | Yes | Yes | Yes | Yes | Yes | Yes | Yes |
| **Was the intervention(s) or treatment procedure(s) clearly described?** | Yes | No | Yes | Yes | No | Yes | Yes | Yes |
| **Was the post-intervention clinical condition clearly described?** | Yes | Yes | Yes | Yes | Yes | Yes |  | Yes |
| **Were adverse events (harms) or unanticipated events identified and described?** | Yes | No | No | No | No | No | No | No |
| **Does the case report provide takeaway lessons?** | Yes | Yes | Yes | Yes | No | Yes | Yes | No |
| **Overall appraisal: Include?** | Yes | Yes | Yes | Yes | Yes | Yes | Yes | Yes |

| **Questions** | **Leung et al. 2022** | **Maejima et al. 2016** | **Mandaliya et al. 2012** | **Meka et al. 2017** | **Nieves et al. 2013** | **Paparoupa et al. 2017** | **Paramsothy et al. 2023** | **Pizzuti et al. 2019** |
| --- | --- | --- | --- | --- | --- | --- | --- | --- |
| **Were patient demographic characteristics clearly described?** | Yes | Yes | Yes | Yes | Yes | Yes | Yes | Yes |
| **Was the patient history clearly described and presented as a timeline?** | Yes | Yes | Yes | No | Yes | Yes | No | Yes |
| **Was the current clinical condition of the patient on presentation clearly described?** | Yes | Yes | Yes | Yes | Yes | Yes | Yes | Yes |
| **Were diagnostic tests or assessment methods and the results clearly described?** | Yes | Yes | Yes | No | Yes | Yes | Yes | Yes |
| **Was the intervention(s) or treatment procedure(s) clearly described?** | Yes | Yes | Yes | No | Yes | Yes | No | Yes |
| **Was the post-intervention clinical condition clearly described?** | Yes | Yes | No | Yes | Yes | Yes | No | Yes |
| **Were adverse events (harms) or unanticipated events identified and described?** | No | No | Yes | No | No | Yes | No | Yes |
| **Does the case report provide takeaway lessons?** | Yes | Yes | No | No | No | No | No | Yes |
| **Overall appraisal: Include?** | Yes | Yes | Yes | Yes | Yes | Yes | Yes | Yes |

| **Questions** | **Prevallet et al. 2021** | **Reddy et al. 2014** | **Singhal et al. 2021** | **Sloan et al. 2016** | **Tayel et al. 2018** | **Townsend et al. 2016** | **Wojas et al. 2021** | **Yost et al. 2022** |
| --- | --- | --- | --- | --- | --- | --- | --- | --- |
| **Were patient demographic characteristics clearly described?** | Yes | Yes | Yes | Yes | Yes | Yes | Yes | Yes |
| **Was the patient history clearly described and presented as a timeline?** | No | Yes | Yes | Yes | Yes | Yes | Yes | Yes |
| **Was the current clinical condition of the patient on presentation clearly described?** | Yes | Yes | Yes | Yes | Yes | Yes | Yes | Yes |
| **Were diagnostic tests or assessment methods and the results clearly described?** | Yes | Yes | Yes | Yes | No | No | Yes | Yes |
| **Was the intervention(s) or treatment procedure(s) clearly described?** | No | Yes | Yes | Yes | No | Yes | Yes | No |
| **Was the post-intervention clinical condition clearly described?** | Yes | Yes | No | Yes | Yes | Yes | Yes | Yes |
| **Were adverse events (harms) or unanticipated events identified and described?** | No | Yes | No | No | No | No | Yes | No |
| **Does the case report provide takeaway lessons?** | No | No | No | Yes | Yes | Yes | Yes | No |
| **Overall appraisal: Include?** | Yes | Yes | Yes | Yes | Yes | Yes | Yes | Yes |

| **Questions** | **Young et al. 2021** | **Zhang et al. 2016** |
| --- | --- | --- |
| **Were patient demographic characteristics clearly described?** | Yes | Yes |
| **Was the patient history clearly described and presented as a timeline?** | Yes | Yes |
| **Was the current clinical condition of the patient on presentation clearly described?** | Yes | Yes |
| **Were diagnostic tests or assessment methods and the results clearly described?** | Yes | Yes |
| **Was the intervention(s) or treatment procedure(s) clearly described?** | No | Yes |
| **Was the post-intervention clinical condition clearly described?** | Yes | Yes |
| **Were adverse events (harms) or unanticipated events identified and described?** | No | No |
| **Does the case report provide takeaway lessons?** | No | Yes |
| **Overall appraisal: Include?** | Yes | Yes |

**Johanna Briggs Institute Critical Appraisal Checklist for Case Series**

| **Questions** | **Lee et al. 2022** | **Jacob et al. 2021** | **Jideh et al. 2016** | **Lavette et al. 2023** | **Schoepfer et al. 2024** |
| --- | --- | --- | --- | --- | --- |
| **Were there clear criteria for inclusion in the case series?** | Yes | Yes | Yes | Yes | Yes |
| **Was the condition measured in a standard, reliable way for all participants included in the case series?** | Yes | Yes | Yes | Yes | Yes |
| **Were valid methods used for identification of the condition for all participants included in the case series?** | Yes | Yes | Yes | Yes | Yes |
| **Did the case series have consecutive inclusion of participants?** | Yes | Yes | Yes | No | No |
| **Did the case series have complete inclusion of participants?** | Yes | No | Yes | No | No |
| **Was there clear reporting of the demographics of the participants in the study?** | Yes | Yes | Yes | Yes | Yes |
| **Was there clear reporting of clinical information of the participants?** | Yes | Yes | Yes | Yes | Yes |
| **Were the outcomes or follow up results of cases clearly reported?** | Yes | No | Yes | Yes | Yes |
| **Was there clear reporting of the presenting site(s)/clinic(s) demographic information?** | Yes | No | Yes | Yes | Yes |
| **Was statistical analysis appropriate?** | Yes | Yes | Yes | Yes | Yes |
| **Overall appraisal: Include?** | Yes | Yes | Yes | Yes | Yes |
